# Two-cationic 2-methylbenzothiazole derivatives as green light absorbed sensitizers in initiation of free radical polymerization

**DOI:** 10.1007/s00396-015-3572-1

**Published:** 2015-04-03

**Authors:** Janina Kabatc, Katarzyna Kostrzewska, Katarzyna Jurek

**Affiliations:** Faculty of Chemical Technology and Engineering, UTP University of Science and Technology, Seminaryjna 3, 85-326 Bydgoszcz, Poland

**Keywords:** Photosensitizer, Photoinitiator, Radical polymerization, Kinetic

## Abstract

*N*-Methylpyridinium esters derivatives of 2-methylbenzothiazole hemicyanine dyes photoinitiators/photosensitizers derived from *N*-propyl-3-[*N*-2-methylbenzothiazolo]-4-pyridyno phenylacetic acid ester diiodide and *N*-propyl-3-[*N*-2-me]thylbenzothiazolo]-4-pyridino diphenylacetic acid ester diiodide were synthesized and proposed as new photoinitiators of polymerization of 2-ethyl-(2-hydroxymethyl)-1,3-propanediol triacrylate under argon laser exposure at 514 nm. These compounds exhibit a strong absorption around 520 nm. The dye/borate salt, dye/borate salt/*N*-methoxypyridinium salt, dye/borate salt/diphenyliodonium salt, and dye/borate salt/1,3,5-triazine derivative combinations are very efficient in initiating of radical photopolymerization of triacrylate. Excellent polymerization profiles were obtained. The effect of both sensitizer and co-initiator structure on the ability to initiate of free radical polymerization of photoinitiating systems was also presented. The mechanism was discussed for different multicomponent initiating systems.

**Graphical Abstract:**
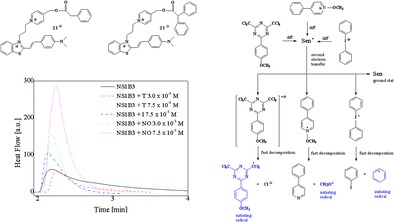
Two-cationic 2-methylbenzothiazole derivatives as green light absorbed sensitizers in initiation of free radical polymerization.

Two-cationic 2-methylbenzothiazole derivatives as green light absorbed sensitizers in initiation of free radical polymerization.

## Introduction

Among different polymer synthesis approaches, the free radical photopolymerization is a fast and efficient method for the production of polymeric materials under light irradiation. It is based on the use of photoinitiating systems suitable to absorb UV or visible light radiations at appropriate wavelengths and to produce primary active species (radicals, cations, and acid) being able to transform multifunctional monomers (acrylates or epoxides) and prepolymers into highly crosslinked networks [1–3]. Three-component photoinitiating system (PIS) were used to achieve a highly efficient PIS for visible light free radical polymerization. Generally, three-component photoinitiating systems consist of a light absorbing dye (sensitizer S), an electron acceptor (A), and an electron donor (D) [2]. In such systems, upon irradiation, photoinduced electron transfer reaction between the dye and one of the components (for example, A) give rise to an initiating radical (R^•^) after decomposition of A^•−^, which then react with monomer to form growing polymer chain. In a following second process, the resulting oxidized dye intermediate S^•+^ interacts with another component (donor, D), which leads to additional initiating radicals (R′^•^) and regenerates the dye in the ground state. The sensitizer is then newly available to absorb light and run into a new cycle. In such photoinitiating systems two initiating radicals are produced within one cycle. Both features are responsible for the higher efficiency of photocyclic initiating systems toward free radical polymerization [2].

In almost all cases of three-component systems, electron donating compounds are designed to decompose after electron transfer in order to prevent unwanted back electron transfer in the (D^•−^S^•+^) dye-donor contact ion pair. This side reaction gives back the reactants and is a loss of energy. Decomposable electron acceptors also prevent bimolecular radical ion recombination after electron transfer. Those unstable electron acceptors decompose or dissociate after electron transfer and generate radicals. Thus, those systems provide excellent results because these acceptors not only prevent undesirable recombination process but also produce new secondary radicals, which enhance the photoreactivity toward free radical polymerization [4].

The three-component photoinitiating systems possess many advantages, such as (i) a better absorption of PIS, (ii) an enhanced photochemical and chemical reactivity, (iii) a higher practical efficiency (in terms of polymerization rates and conversion) [5], (iv) the possibility for either a tunable absorption through the choice a well-designed class of PI or a selective excitation at a given laser line, (v) the promising design of systems less sensitive to oxygen, (vi) the development of dual radical/cationic PISs, and (vii) the proposal of photoinitiators behaving as photocatalysts [6].

Many of the same photosensitizers used for two-component electron-transfer initiating systems may also be used in three-component ones [7, 8]. Examples include coumarin dyes, xanthene dyes, acridine dyes, thiazole dyes, thiazine dyes, oxazine dyes, azine dyes, aminoketone dyes, porphyrins, aromatic polycyclic hydrocarbons, *p*-substituted aminostyryl ketone compounds, aminotriaryl methanes, merocyanines, and pyridinium dyes [8–14].

The development of new photoinitiating systems remains an interesting challenge. In specific areas, for example, in graphic arts or in conventional clear coat and overprint varnish applications, the photoinitiators must exhibit particular properties, among them a high photochemical reactivity leading to high curing rates.

Therefore, the objective of this work is to provide a new method for the improvement of visible light free radical photoinitiating systems, having high efficiency. Our system consist of a dye (**NS**), an electron donating radical generator (**B**), and an unstable dissociative electron acceptor (**NO**, **I**, or **T**).

Taking into account earlier works in this area [15, 16], we explore here the possibility of using four *N*-methylpyridinium esters derivatives of 2-methylbenzothiazole hemicyanine dyes that could exhibit a broad visible absorption and act as photosensitizers of free radical polymerization operating in a visible light region.

Similar sensitizers paired with alkyltriphenylborates are very often used as a dyeing photoinitiating systems for free radical polymerization of acrylates. Unfortunately, their photoinitiating ability is lower than that of other photoinitiators commonly used in industry. To improve it, many kinds of compounds have been added to such photoinitiating systems, for example, *N*-alkoxypyridinium salts [17, 18], 1,3,5-triazine derivatives or *N*-methylpyridinium derivatives [5, 19]. In our previous papers [5, 15], it was shown that the *N*-methylpyridinium derivatives can act as an efficient second co-initiator for free radical polymerization induced by cyanine dye, a popular photosensitizer. The second co-initiator strongly affects the rates of cyanine/borate-induced polymerization. It was found that the second electron transfer process from the dye-based radical to the *N*-methylpyridinium derivative enhances the rate of photopolymerization. Generally, by a combination of *N*-methylpyridinium derivative and cyanine borate salt, two radicals can be generated per one absorbed photon, thus enhancing the overall efficiency of polymerization.

**Scheme 1:**
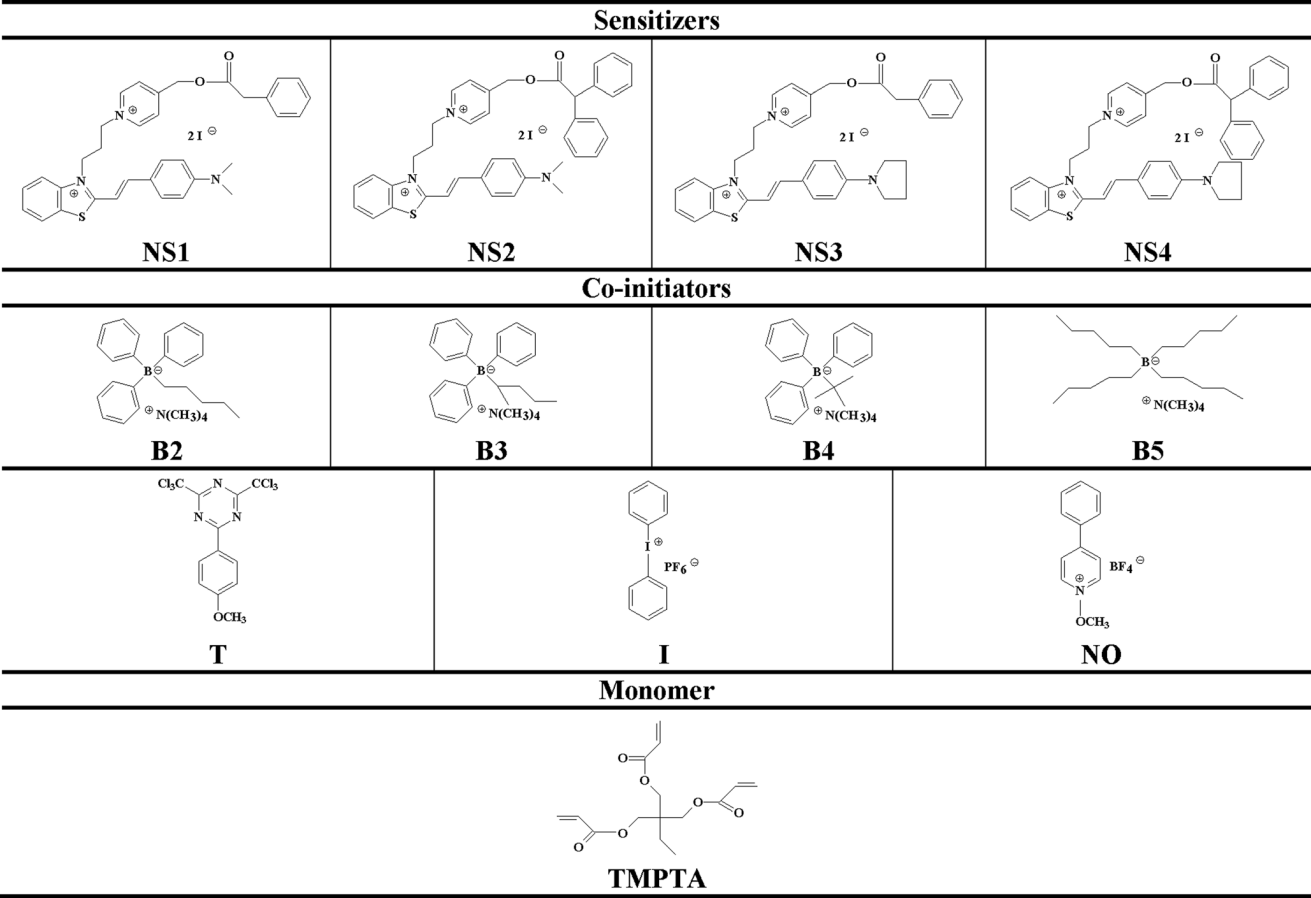
Structures of sensitizers, co-initiators, and monomer

Our actual search for new initiating systems for visible light prompts us to develop new dyes based on the *D*–*π*–*A* structure. They are based on the difunctional structures where benzothiazole moiety is linked to *N*-alkyl methylpyridinium ester group through a carbon–carbon bond and is extension of our previous studies [20]. Therefore, present paper is focused on the synthesis and application of the visible light bifunctional two-component photoinitiating systems composed of sensitizer with phenylacetic acid or diphenylacetic acid (*N*-methylpyridinium)methyl ester group attached to the nitrogen atom in heterocyclic ring (dyes **NS1**, **NS2**, **NS3**, and **NS4**) and borate salts (**B2**, **B3**, **B4**, and **B5**), as well as three-component systems composed of 2-(4-methoxystyryl)4,6-bis(trichloromethyl)-1,3,5-triazine (**T**), diphenyliodonium hexafluorophosphate (**I**) or *N*-methoxy-4-phenylpyridinium tetrafluoroborate (**NO**) in photopolymerization reaction of triacrylate (TMPTA) (Scheme 1).

## Experimental

### Materials

The substrates used for the synthesis of dyes [2-methylbenzothiazole, 4-pirydylcarbinol, diiodopropane, trimethylamine, phenylacetic acid chloride, *N*,*N*-dicycloheksacarbodiimide, diphenylacetic acid, *p*-(*N*,*N*-dimethylamino)benzaldehyde, *p*-pyrrolidinobenzaldehyde], 2-ethyl-2-(hydroxymethyl)-1,3-propanediol triacrylate [trimethylolpropane triacrylate (TMPTA)], and 1-methyl-2-pyrrolidinone (MP) were purchased from Aldrich (Poland) and were used without further purification. The purity of all chemical used was as required for spectroscopic studies (≥99 %).

### Synthesis of photosensitizers

**Scheme 2:**
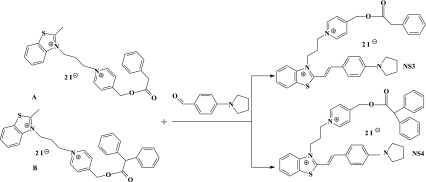
The synthesis of photosensitizers (**NS3**) and (**NS4**)

The investigated photosensitizers (**NS1**, **NS2**, **NS3**, and **NS4**) shown in Scheme 1 were prepared in multistep reactions as was described in our previous paper [20]. The condensation presented in Scheme 2 is a last step of synthesis of dyes **NS3** and **NS4**. Details of synthesis of sensitizers **NS1** and **NS2** are presented in literature [20] and are not repeat here.

The synthesis of 2-methylbenzothiazole quaternary salts: *N*-propyl-3-[*N*-2-methylbenzothiazolo]-4-pyridyno phenylacetic acid ester diiodide (A) and *N*-propyl-3-[*N*-2-methylbenzothiazolo]-4-pyridyno diphenylacetic acid ester diiodide (B), as well as dyes **NS1** and **NS2** were earlier described by us [20]. In the present paper, the synthesis of two sensitizers is presented.

These photosensitizers (**NS1**, **NS2**, **NS3**, and **NS4**) were prepared by Knoevenagel condensation of appropriate 2-methylbenzothiazole quaternary salt with corresponding benzaldehyde.

The synthesis of phenylacetic acid (4-pyridyno)methyl ester and diphenylacetic acid (4-pyridyno)methyl ester was carried out based on the method described by Sunderarajan [21].

*n*-Butyltriphenylborate tetramethylammonium salt (**B2**), *sec*-butyltriphenylborate tetramethylammonium salt (**B3**), *tert*-butyltriphenylborate tetramethylammonium salt (**B4**), and tetrakis (*n*-butylborate) tetramethylammonium salt (**B5**) were synthesized based on the method described by Damico [22].

All compounds were prepared with analytical purity up to accepted standards for new organic compounds (>98 %), which was checked by proton (^1^H) and carbon (^13^C) nuclear magnetic resonance (NMR) spectroscopy and elemental analysis. The spectra obtained were the evidence that the reaction products were of the desired structure.

### Synthesis of dye (**NS3**): the condensation of *N*-propyl-3-[*N*-2-methylbenzothiazolo]-4-pyridyno phenylacetic acid ester diiodide with *p*-pyrrolidinobenzaldehyde

One gram of *N*-propyl-3-[*N*-2-methylbenzothiazolo]-4-pyridyno phenylacetic acid ester diiodide (1.0 mmol) and 0.26 g of *p*-pyrrolidinobenzaldehyde (1.0 mmol) were suspended in methanol (40 mL), and a few drops of piperidine were added. Immediately, the solution turned deep red. The solution was refluxed for 6 h. After cooling dark violet solid was filtered off, crystallized form ethanol and dried on the air.

Yield: 69.71 %, m.p. 252–254 °C.

^1^H NMR (DMSO-*d*_*6*_), δ (ppm): 2.001 (s, 4H, –CH_2_O); 2.375 (m, 2H, –CH_2_); 3.335–3.433 (m, 8H, –CH_2_); 4.763 (m, 4H, N–CH_2_); 6.700–6.743 (d, 4H, Ar); 7.486–7.565 (m, 1H, Ar, 1H, –CH=CH–); 7.623–7.816 (t, 4H, Ar; 1H –CH=CH–); 7.894–7.938 (d, 4H, Ar); 8.052–8.127 (m, 2H, Ar); 8.270–8.310 (d, 2H, Pyr). ^13^C NMR (DMSO-*d*_*6*_), δ (ppm): 2.252; 24.863; 32.309; 33.784; 47.665; 48.293; 96.291; 104.766; 112.384; 115.334; 121.132; 123.826; 133.138; 141.058; 150.761; 151.061; 171.169; 221.206. Anal. Calcd for: C_36_H_37_N_3_SO_2_I_2_: C, 52.11; H, 4.46; N, 5.07. Found: C, 51.99; H, 4.40; N, 5.17.

### Synthesis of dye (**NS4**): the condensation of *N*-propyl-3-[*N*-2-methylbenzothiazolo]-4-pyridyno diphenylacetic acid ester diiodide with *p*-pyrrolidinobenzaldehyde

One gram of *N*-propyl-3-[*N*-2-methylbenzothiazolo]-4-pyridyno diphenylacetic acid ester diiodide (1.0 mmol) and 0.23 g of *p*-pyrrolidinobenzaldehyde (1.0 mmol) were suspended in methanol (50 mL), and a few drops of piperidine were added. Immediately, the solution turned deep red. The solution was refluxed for 6 h. After cooling, dark violet solid was filtered off, crystallized form ethanol, and dried on the air.

Yield: 66.12 %, m.p. 247 °C.

^1^H NMR (DMSO-*d*_*6*_), δ (ppm): 2.007 (s, 2H, –CH_2_O); 2.201 (s, 2H, –CH_2_); 3.331–3.439 (m, 8H, –CH_2_); 4.772 (m, 4H, N–CH_2_); 6.693-6.737 (d, 2H, –CH=CH–); 7.347 (s, 2H, Ar); 7.492–7.567 (t, 1H Ar); 7.629–7.820 (m, 10H, Ar); 7.898–7.942 (d, 4H, Ar); 8.056–8.129 (t, 4H, Ar); 8.274–8.314 (d, 2H, Pyr). ^13^C NMR (DMSO-*d*_*6*_), δ (ppm): 2.261; 24.863; 47.438; 47.656; 53.327; 112.111; 112.384; 121.132; 128.396; 128.523; 134.358; 151.070; 171.169. Anal. Calcd for: C_42_H_41_N_3_SO_2_I_2_: C, 55.69; H, 4.53; N, 4.64. Found: C, 55.71; H, 4.60; N, 4.57.

### Synthesis of borate salts of hemicyanine dyes

In the course of this work, it was necessary to exchange the counterion generated in the synthesis of hemicyanine dyes on *n*-, *tert*-, *sec*-butyltriphenylborate anion, and tetra-*n*-butylborate anion. Therefore, the substitution reaction was the last step of the synthesis. The exchange of iodide anion on borate anion transfers these dyes into efficient photoinitiating systems for free radical polymerization. Synthesis of these compounds was carried out using procedure described by Schuster et al. [23].

### Techniques

#### Spectroscopic measurements

Ultraviolet–visible (UV–Vis) electronic absorption spectra were obtained using a Shimadzu UV–Vis Multispec-1500 spectrophotometer (Japan), and steady-state fluorescence was performed using a FLS920 Edinburgh Instruments Spectrophotometer (England). The ^1^H and ^13^C NMR spectra were recorded with the use of a Varian Gemini 200 spectrometer operating at 200 MHz. Dimethylsulfoxide (DMSO-*d*_*6*_) was used as a solvent and tetramethylsilane as internal standard.

The elemental analysis was made with a Vario MACRO 11.45-0000, Elementar Analyser System GmbH (Germany), operating with the software VARIOEL 5.14.4.22.

#### Reduction and oxidation potentials

The reduction and oxidation potentials were measured by cyclic voltammetry. An Electroanalytical MTM System model EA9C-4z (Poland), equipped with a small-volume cell, was used for the measurements. A 1-mm platinum electrode was applied as the working electrode. A platinum wire constituted the counter electrode, and a silver/silver chloride (Ag/AgCl) electrode served as a reference. The supporting electrolyte was 0.1 M tetrabutylammonium perchlorate in dry acetonitrile. The solution was deoxygenated by bubbling argon gas through the solution. The potential was swept from −1.8 to 1.8 V at a rate of 500 mV/s to record the current–voltage curve.

#### Polymerization

Photoinitiated polymerization rate (*R*_p_) profiles were determined by differential scanning calorimetry (DSC) under isothermal conditions at room temperature using a photo-DSC apparatus constructed on the basis of a TA Instruments DSC 2010 Differential Scanning Calorimeter (USA). The sample (0.035 ± 0.002 g) was polymerized in open aluminum pans with a diameter of 6.6 mm. Irradiation of the polymerization mixture was carried out using the visible emission (514 nm) of the air-cooled Ion Laser Systems model 177-G01 (Spectra-Physics, USA). The average power of irradiation was 15 mW/0.196 cm^2^ at 514 nm. The light intensity was measured by a Coherent Model Fieldmaster power meter (Germany).

The polymerization solution was composed of 1 mL of MP and 9 mL of TMPTA. The photoinitiator concentrations used in the experiments were in the range from 1.0 × 10^−4^ M to 7.5 × 10^−3^ M. As a reference sample, a polymerizing mixture containing dye halide (sensitizer without a co-initiator) was used. The polymerizing mixture was not deaerated. In order to reduce the effect of diffusion-controlled termination, the effect of a network formation, the Norrish–Troomsdorff effect, and the radicals trapping effect, the initial rates of polymerization were taken into account for further consideration.

The degree of conversion of the monomer into polymer determined after a specific irradiation time was also presented here. The conversion percentages were obtained integrating the area under the exothermic peak

1 $$ C\%=\frac{\varDelta {H}_t}{\varDelta {H}_0}\times 100 $$

where Δ*H*_*t*_ is the reaction heat evolved at time *t*, and Δ*H*_0_ is the heat evolved assuming total conversion. Polymerization rates were calculated using Eq. 2

2 $$ {R}_{\mathrm{p}}=\frac{\mathrm{d}H/\mathrm{d}t}{\varDelta {H}_0} $$

## Results and discussion

### Absorption properties of dyes (**NS1**, **NS2**, **NS3**, and **NS4**)

**Fig. 1:**
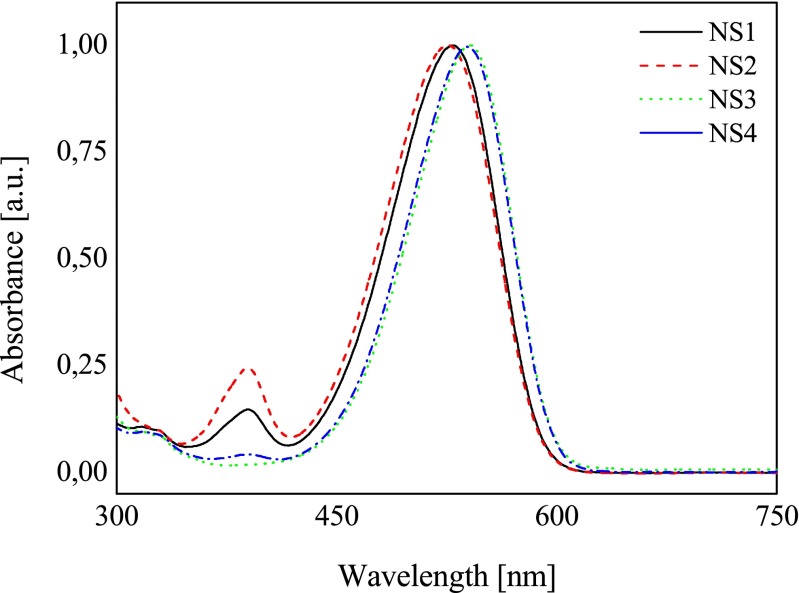
Electronic absorption spectra of dyes studied in acetonitrile recorded at 293 K

The sensitizers differ in the type of substituent in *p*-position of styryl moiety and the type of *N*-methylpyridinium group attached to the nitrogen atom of benzothiazolium ring. The ground state absorption spectra of *N*-methylpyridinium esters derivatives of 2-methylbenzothiazole hemicyanine dyes allow a large and efficient matching with the light source emission spectra (argon laser at 514 nm) (Fig. 1).

Remarkably, high molar extinction coefficients in acetonitrile solution are determined (about 80 000 M^−1^ cm^−1^ for **NS1**, 70 000 M^−1^ cm^−1^ for **NS2**, 100 000 M^−1^ cm^−1^ for **NS3**, and 93 000 M^−1^ cm^−1^ for **NS4**, respectively) compared to absorption only in the UV range for 2-methylbenzothiazole, *p*-(substituted)benzaldehyde, *N*-methylpyridine.

**Fig. 2:**
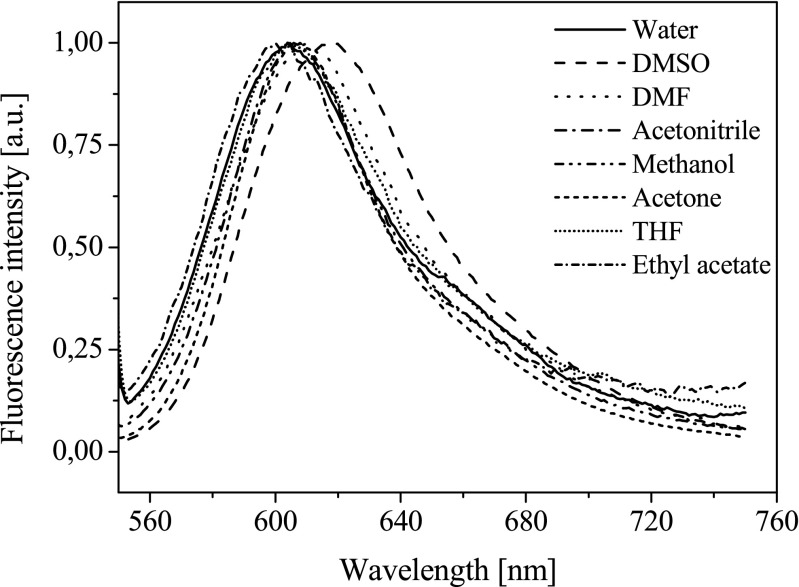
Influence of the solvent polarity on the fluorescence spectra of dye **NS1** recorded at 293 K (the excitation wavelength, *λ*_EX_ = 530 nm)

These dyes can be considered as push-pull aromatic chromophores. The unsymmetrically substituted *D*–*π*–*A* arrangements bearing electron donor (*D*) and electron acceptor (*A*) functionalities at both ends of a planar conjugated spacer are well-known to feature a large change in their absorption spectra and/or emission spectra as a function of the solvent polarity [24]. A solvatochromic study was carried out for all new dyes in eight different polarity solvents: water, DMSO, *N*,*N*-dimethylformamide (DMF), acetonitrile, methanol, acetone, tetrahydrofuran (THF), and ethyl acetate. Figure 2 presents the influence of polarity of solvent on the emission spectra of selected dye.

The solvent polarity only slightly effects on the spectroscopic properties of dyes studied. The value of Stokes shift is about 2000 cm^−1^.

The spectroscopic parameters confirm that an important charge transfer occurs in the electronic transition. It is well known that in a case of unsymmetrical dyes the lowest energy transition involves strongly delocalized HOMO and LUMO orbitals. For these dyes, the HOMO and LUMO orbitals are mainly localized on amine and benzothiazole fragment, respectively. The results obtained are in good agreement with those reported for similar hemicyanine dyes [25]. This highlights a charge transfer character in agreement with the push–pull character of this structure.

It should be noted that no direct relationships were found with the empirical solvatochromic scales evidencing a behavior more complex than that recently encountered in other push–pull systems [26].

### Free radical polymerization

#### Two-component photoinitiating systems

The photoinitiating systems composed of hemicyanine dyes (**NS1**, **NS2**, **NS3**, and **NS4**) as photosensitizer with various co-initiators (borate salts) (structures given in Scheme 1) were used for the initiation of free radical polymerization. The polymerization solution consisted of sensitizer as borate salts with concentration in the range from 1.0 × 10^−4^ M to 7.5 × 10^−3^ M. The polymerization process was initiated by irradiation at 514 nm. As it was shown above, at this wavelength only sensitizer absorbs the light.

#### Influence of photoinitiator concentration on the rate of polymerization

**Fig. 3:**
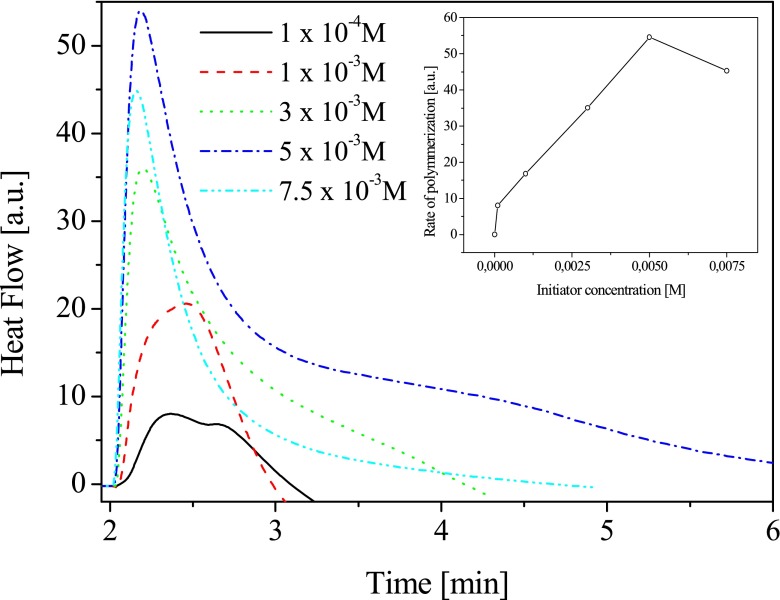
Rate of polymerization versus selected photoinitiator (**NS1B2**) concentrations

In order to optimize the composition of a polymerization mixture, at the beginning, the influence of photoinitiator concentration on observed rate of polymerization was determined. Figure 3 presents the relationship between the initial rate of polymerization and concentration of photoinitiator.

It is evident that, as the photoinitiator concentration is increasing, the initial rate of polymerization increases and reaches a maximum followed by a continuous mild decrease. For the tested two-component photoinitiating systems, under experimental conditions, the highest rate of polymerization was observed at the initiator concentration of about 5.0 × 10^−3^ M. The photoinitiator concentration also effects on the degree of monomer conversion. The monomer conversion observed under experimental conditions was in range from 1.11 to 12.01 %. The highest values was obtained for photoinitiator concentration equal 5.0 × 10^−3^ M. The reduction of the photoinitiated polymerization rate at higher initiator concentration can by easily understood taking into account the decrease in the penetration depth of the laser beam across the polymerizing formulation layer [27]. Thus, all kinetic measurements were carried out at photoinitiator concentration equal 5.0 × 10^−3^ M.

#### Influence of photoinitiator structure on the rate of polymerization

**Fig. 4:**
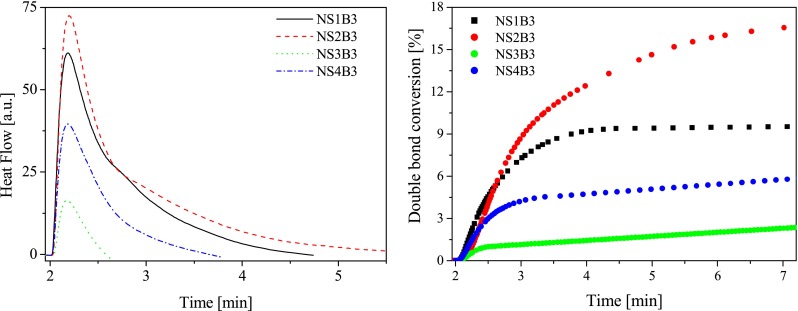
*Left* The family of kinetic curves recorded during the measurements of the flow of heat for the photoinitiated polymerization of the TMPTA/MP (9/1) mixture initiated by systems composed of new sensitizer as *sec*-butyltriphenylborate salt (**B3**). The concentration of photoinitiating systems was equal 5.0 × 10^−3^ M, *I*_a_ = 76 mW × cm^−2^. *Right* Relationship between the degree of double bond (–CH=CH–) conversion of trimetylolpropane triacrylate and irradiation time. The type of photoinitiating system is marked in the figure

**Fig. 5:**
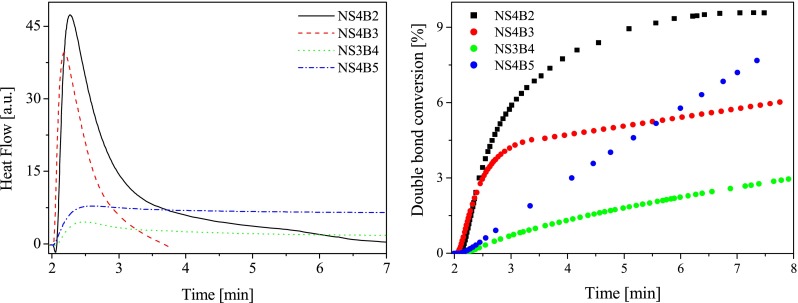
*Left* The family of kinetic curves recorded during the measurements of the flow of heat for the photoinitiated polymerization of the TMPTA/MP (9/1) mixture initiated by systems composed of sensitizer **NS3** or **NS4** and *n*-butyltriphenylborate salt, *sec*-butyltriphenylborate salt, *tert*-butyltriphenylborate salt (**B2**, **B3**, **B4**), and tetra-*n*-butylborate salt (**B5**). The concentration of photoinitiating systems was equal 5.0 × 10^−3^ M, *I*_a_ = 76 mW × cm^−2^. *Right* Relationship between the degree of double bond (–CH=CH–) conversion of trimetylolpropane triacrylate and irradiation time. The type of photoinitiating system is marked in the figure

The influence of both sensitizer and co-initiator structure on the photoinitiating ability of new photoinitiators is shown in Figs. 4 and 5.

A relatively low final conversion is obtained when using new sensitizer paired with *sec*-butyltriphenylborate or *tert*-butyltriphenylborate anions as an electron donor (Figs. 4 and 5), whereas better polymerization profiles are reached for following photoinitiating systems: **NS1B3**, **NS2B3**, **NS3B2**, and **NS4B2** (final conversion above 10 %, Figs. 4 and 5). It should be noted that their photoinitiating ability is lower than well-known commonly used photoinitiators operating in the visible light region [3, 4, 24, 26]. From results obtained, it is known that all sensitizers in presence of borates **B4** and **B5** are not found to be efficient sensitizers for free radical polymerization of acrylate tested. The reactivity of sensitizers and borate salts studied will be discussed below.

#### Three-component photoinitiating systems

**Fig. 6:**
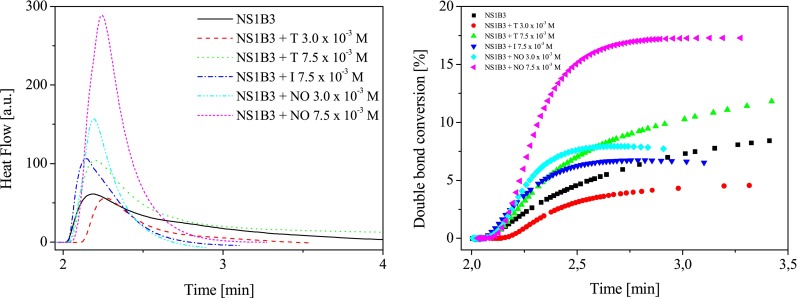
*Left* The family of kinetic curves recorded during the measurements of the flow of heat for the photoinitiated polymerization of the TMPTA/MP (9/1) mixture initiated by two-component system composed of sensitizer **NS1** and *sec*-butyltriphenylborate salt (**B3**) in presence of 1,3,5-triazine derivative (**T**), iodonium salt (**I**), and *N*-methoxypyridinium salt (**NO**), respectively. The concentration of photoinitiating systems was equal to 5.0 × 10^−3^ M, and second co-initiators is marked in the figure, *I*_a_ = 76 mW × cm^−2^. *Right* Relationship between the degree of double bond (–CH=CH–) conversion of trimetylolpropane triacrylate and irradiation time. The type of photoinitiating system is marked in the figure

As it is seen from the kinetic results presented in Figs. 4 and 5, the quite slow monomer conversion is obtained when using hemicyanine borate photoinitiating systems, whereas a very fast rate of polymerization and higher conversions of monomer are reached with some three-component photoinitiating systems. Figure 6 shows the photopolymerization results for the three-component systems containing sensitizer **NS1** as an ion pair with *sec*-butyltriphenylborate anion.

The rates of radical polymerization initiated by two- and three-component photoinitiating systems are presented in Table 1.

**Table 1:** Thermodynamic and kinetic parameters of selected two- and three-component photoinitiating systems under study

Photoinitiating system	Δ*G*_el_ (eV)	*R*_p_ (μmol/s)	Monomer conversion (%)^a^	Polymerization quantum yield
NS1B2	0.45	0.413	12.0	4.84
NS1B3	0.255	0.79	9.6	9.29
NS2B2	0.24	0.4	2.5	4.66
NS2B3	0.045	0.93	16.7	10.86
NS3B2	0.225	0.55	6.0	6.40
NS3B3	0.03	0.21	2.4	2.46
NS3B4	−0.019	0.06	3.0	0.68
NS4B2	0.35	0.605	9.5	7.10
NS4B3	0.155	0.506	5.9	5.93
NS4B5	−0.159	0.1	7.8	1.17
NS1B3 + T C_1_		0.72	6.1	8.43
NS1B3 + T C_2_		1.33	21.0	15.55
NS1B3 + I C_1_		1.36	8.95	15.99
NS1B3 + I C_2_		0.33	2.5	3.85
NS1B3 + NO C_1_		2.01	8.11	23.57
NS1B3 + NO C_2_		3.71	17.45	43.49

^a^Determined after 7 min of irradiation

As shown in Table 1, the rate of polymerization initiated by three-component systems containing **NS1B3** and 2-(4-methoxystyryl)4,6-bis(trichloromethyl)-1,3,5-triazine (**T**), diphenyliodonium hexafluorophosphate (**I**) or *N*-methoxy-4-phenylpyridinium tetrafluoroborate (**NO**) as second co-initiators showed about two to five times higher sensitivity than that of corresponding two component system (**NS1B3**). The photoinitiating systems containing *N*-methoxypyridinium salt showed a slight increase in rate of photopolymerization; however, the effect is smaller when 2-(4-methoxystyryl)4,6-bis(trichloromethyl)-1,3,5-triazine (**T**) or diphenyliodonium hexafluorophosphate (**I**) is added.

#### Electron transfer between sensitizer excited state and co-initiators

Generally, the values of the Gibbs free energy change Δ*G*_et_ for photoinduced electron transfer are given by the Rehm–Weller equation (Eq. 3) [28]:

3 $$ \varDelta {G}_{\mathrm{et}}={E}_{\mathrm{ox}}\hbox{--} {E}_{\mathrm{red}}\hbox{--} {E}^{*} + C $$

where *E*_ox_ and *E*_red_ are the half-wave oxidation and reduction potentials for the donor and the acceptor, respectively. E* stands for the energy of the excited state. The Coulombic term *C* is usually neglected in polar solvent.

The irradiation of two-component photoinitiating systems with argon laser at 514 nm causes a fast bleaching of sensitizer. It results (4) from the oxidation of borate salt (**B2**, **B3**, **B4**, or **B5**) by sensitizer leading to sensitizer-based radical, triphenylboron (or tri-*n*-butylboron) and butyl radical Bu^•^ (originating from the subsequent very fast carbon-boron bond cleavage in boranyl radical (5) [5, 20].

4 $$ \begin{array}{l}\mathrm{S}\mathrm{e}\mathrm{n}\overset{\mathrm{h}\upnu}{\to }{}^1\mathrm{S}\mathrm{e}\mathrm{n}\hfill \\ {}{}^1\mathrm{S}\mathrm{e}\mathrm{n}+\mathrm{B}\mathrm{u}\mathrm{B}{{\left(\mathrm{P}\mathrm{h}\right)}_3}^{-}\to {\mathrm{Sen}}^{\bullet }+\mathrm{B}\mathrm{u}\mathrm{B}{{\left(\mathrm{P}\mathrm{h}\right)}_3}^{\bullet}\hfill \end{array} $$

or

5 $$ \begin{array}{l}{}^1\mathrm{S}\mathrm{e}\mathrm{n}+{\mathrm{B}\mathrm{u}}_4{\mathrm{B}}^{-}\to {\mathrm{Sen}}^{\bullet }+{\mathrm{B}\mathrm{u}}_4{\mathrm{B}}^{\bullet}\hfill \\ {}\mathrm{B}\mathrm{u}\mathrm{B}{{\left(\mathrm{P}\mathrm{h}\right)}_3}^{\bullet}\to {\mathrm{B}\mathrm{u}}^{\bullet }+{\left(\mathrm{P}\mathrm{h}\right)}_3\mathrm{B}\hfill \\ {}{\mathrm{B}\mathrm{u}}_4{\mathrm{B}}^{\bullet}\to {\mathrm{B}\mathrm{u}}^{\bullet }+{\left(\mathrm{B}\mathrm{u}\right)}_3\mathrm{B}\hfill \end{array} $$

Three different butyl radicals are formed, i.e., *n*-, *sec*-, and *tert*-butyl; therefore, the photoinitiating ability of photoinitiating systems may depend on the reactivity or quantum yield of radical formation. Based on the kinetic results, it is seen that the type of borate salts strongly affects the photoinitiating ability of PIS.

It should be noted that in a case of photoinitiating systems acting via photoinduced electron transfer process, thermodynamic factors also influence on the effectiveness of initiating of polymerization process. Therefore, in the next step, the values of free energy change for photoinduced electron transfer process were calculated.

The values of Δ*G*_et_ were calculated using the oxidation potentials of borate salts (*E*_ox_ = 1.16 , 0.965, 0.916, and 0.651 eV for **B2**, **B3**, **B4**, and **B5**, respectively), the singlet excited state energy [*E*_00_(*S*)] of the dyes (*E*_00_ = 2.16, 2.17, 2.14, and 2.14 eV for **NS1**, **NS2**, **NS3**, and **NS4**, respectively) and the reduction potential *E*_red_(Sen/Sen^•^) of sensitizers (*E*_red_ = −1.45, −1.236, −1.205, and −1.33 eV for **NS1**, **NS2**, **NS3**, and **NS4**, respectively). The Coulombic energy coefficient was omitted in these calculations because the neutral radicals of borate salt as well as sensitizers were formed in the electron transfer process. The electrochemical properties and thermodynamic and kinetic parameters are presented in Table 1.

The intramolecular electron transfer between the excited dye and organoborate salt must be thermodynamically allowed. The values of free energy changes for electron transfer process, calculated from the Rehm–Weller equation range from −0.284 to 0.45 eV.

The data presented in Table 1 clearly show the dependence of the rate of initiation polymerization on the free energy change for electron transfer process. However, the observed effect of free energy change for electron transfer process on the rate of free radical polymerization is opposite to those commonly observed [1, 7, 8, 29]. Therefore, for the photoinitiating systems studied, the observed rate of initiation of polymerization process depends on the thermodynamic conditions. The decrease in the free energy change of electron transfer process causes in an increase in the rate of free radical polymerization of TMPTA.

Therefore, the lower photoinitiating ability observed for the photoinitiating systems possessing more negative values of Δ*G*_et_ can be explained by a back electron transfer in reaction (1) regenerating the starting compounds. Such a deactivation pathway decreases the yield in butyl radical and sensitizer-based radical in line with the very low efficiency of systems containing *tert*-butyltriphenylborate salt (**B4**) and tetra-*n*-butylborate salt (**B5**) in polymerization experiments.

**Scheme 3:**
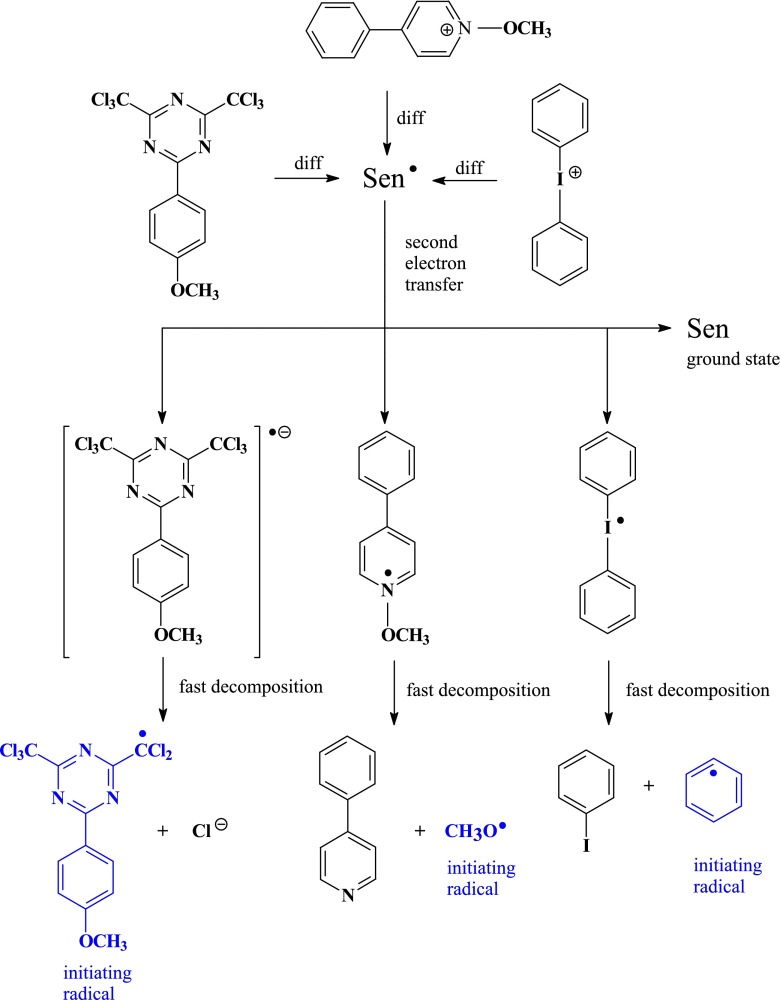
Mechanism of generation of second initiating radicals in three-component photoinitiating systems

On the basis of the photochemistry of borate anion [29, 30], photochemistry of *N*-alkoxypyridinium cation [18], photochemistry of 1,3,5-triazine derivatives [31–33], and photochemistry of iodonium salt [24], as well as results of nanosecond laser flash photolysis described in our previous papers [5, 15, 17, 19], we propose the mechanism of primary and secondary processes occurring in three-component photoinitiating systems (Scheme 3).

After excitation of ion pair composed of electron acceptor (dye) and electron donor (borate salt), the photoinduced electron transfer process takes place. The resulting boranyl radical decomposes, yielding neutral triphenylboron and butyl radical [29, 30]. The other product of electron transfer reaction, dye-based radical, could participate in a second electron transfer process with second co-initiator: *N*-methoxypyridinium cation, 1,3,5-triazine derivative or diphenyliodonium cation.

A word of caution is required for the energy stored in the dye-based radical-second co-initiator pair. For photoinduced electron transfer reaction between a donor and an acceptor, the energy stored in pair is define as the difference between the oxidation potential of a donor and reduction potential of an acceptor (referred as the redox energy). The oxidation potential of the dye-based radical is approximately equal to the reduction potential of the dye cation. The oxidation potentials for the dye-based radical under study are −1.45 V for dye **NS1**, −1.236 for dye **NS2**, −1.205 V for dye **NS3**, and −1.33 V for dye **NS4**, respectively. The reduction potential for *N*-methoxy-4-phenylpyridinium cation is about −0.67 V, for 2-(4-methoxystyryl)4,6-bis(trichloromethyl)-1,3,5-triazine is about −0.84 V, and for diphenyliodonium cation is about −0.2 V, respectively. Thus, the driving force of electron transfer between dye-based radical (**NS1** ^•^) is −0.78 eV for *N*-methoxy-4-phenylpyridinium cation, −0.61 eV for 2-(4-methoxystyryl)4,6-bis(trichloromethyl)-1,3,5-triazine, and −1.25 eV for diphenyliodonium salt, respectively. The negative values indicate that the electron transfer between dye-based radical and all second co-initiators studied is thermodynamically allowed. Basing on the electrochemical measurements and laser flash photolysis results [5, 15, 17], one can conclude that the dye-based radical undergoes one electron oxidation in a presence of second co-initiator. This reaction regenerates the original dye and produces unstable *N*-methoxypyridinium radical, 1,3,5-triazine radical anion, and diphenyliodonium radical as a result of second electron transfer process. In next step, unstable products undergo very fast decomposition leading to the formation: methoxy radical and 4-phenylpyridine, triazinyl radical and halogene anion, and phenyl radical and iodobenzene, respectively. In this way, second initiating radicals are formed (methoxy, triazinyl, and phenyl). The enhanced reaction rate observed in the case of three-component photoinitiating systems is explained in part by fact that the methoxy radical, triazinyl radical, and phenyl radical, unlike dye-based radical are active for initiation of free radical polymerization of triacrylate. In addition, since the ground state of sensitizer is regenerated in this reaction, the initiation rate will be enhanced further. On the basis of the earlier discussion, we suggest that the radical formation reactions proceed as illustrated in Scheme 3.

## Conclusion

In this paper, we showed the synthesis and properties of two-cationic sensitizers, which can be apply in polymer chemistry. The synthesized *N*-methylpyridinium esters derivatives of 2-methylbenzothiazole in combination with *n*-butyltriphenylborate salt or *sec*-butyltriphenylborate salt efficiently initiate the free radical polymerization of triacrylate under argon-ion laser (514 nm). Their photoinitiating ability is lower than well-known commonly used photoinitiators operating in the visible light region.

Practical and very efficient multicomponent photoinitiating systems that are useful for photoinitiation of radical polymerization are also described. This approach considers a systems containing hemicyanine dye as borate salt (electron donor) and second co-initiator as a ground state electron acceptor. In this paper, we have offered kinetic studies and experimental evidence for a proposed reaction mechanism by which active radical centers are produced by three-component systems comprised hemicyanine dye, borate salt, and second co-initiator. The systems under study are of special interest because the cationic nature of sensitizer prevents direct reaction with second co-initiators used, thus simplifying the list of possible reactions. As summarized, the mechanism involves electron transfer from borate salt to the sensitizer as the primary photochemical reaction. Second co-initiator is an electron acceptor, acting to reoxidize the sensitizer-based radical back to its original state and allow it to re-enter the primary photochemical process. This reaction also generates free radicals. The **NS1**/*sec*-butyltriphenylborate salt/*N*-methoxy-4-phenylpyridinium salt (or diphenyliodonium salt or 1,3,5-triazine derivative) three-component photoinitiating system generates free radicals (butyl, methoxy, phenyl, and triazinyl) that can start polymerization reaction. Finally, from a combination of butyltriphenylborate salt and the ground state electron acceptor with suitable sensitizer, two radicals can be generated per one absorbed photon, thus enhancing the overall polymerization efficiency. Efficient monomer conversions using the three-component photoinitiating system under monochromatic light source can be also reached.

The approach presented here might be of interest for the synthesis of suitable visible light sensitive high-speed photoinitiators for free radical polymerization.
